# Monitoring Food Marketing Directed to Portuguese Children Broadcasted on Television

**DOI:** 10.3390/nu15173800

**Published:** 2023-08-30

**Authors:** Marta Figueira, Joana Araújo, Maria João Gregório

**Affiliations:** 1 EPIUnit—Instituto de Saúde Pública, Universidade do Porto, 4050-600 Porto, Portugal; figueira.marta@gmail.com (M.F.); jfaraujo@med.up.pt (J.A.); 2 Faculdade de Ciências da Nutrição e Alimentação, Universidade do Porto, 4150-180 Porto, Portugal; 3 Laboratório para a Investigação Integrativa e Translacional em Saúde Populacional (ITR), Universidade do Porto, 4050-600 Porto, Portugal; 4 Departamento de Ciências da Saúde Pública e Forenses, e Educação Médica—Unidade de Epidemiologia, Faculdade de Medicina da Universidade do Porto, 4200-450 Porto, Portugal; 5 Direção-Geral da Saúde, 1049-005 Lisboa, Portugal

**Keywords:** food advertising, food marketing, television, children, childhood obesity

## Abstract

Children are massively exposed to food marketing through television and other forms of media. Marketing strategies promote unhealthy eating behaviours and contribute to childhood obesity. The main aim of this study was to assess the potential exposure and power of food advertisements aimed at children, broadcasted on Portuguese television. Television data was recorded for two weekdays and two weekend days between 6 am and 10 pm during November 2021 from four free-access Portuguese television channels. Data was analysed according to the World Health Organization television protocol and Portuguese Legislation. We identified 5272 advertisements, of which 11.2% were for food and beverages (*n* = 590). Most advertised food categories for children and adolescents were chocolate and bakery products (42.0%), soft drinks (26.7%), and yoghurt (16.0%), and none met the nutritional profile outlined by the national legislation. Unhealthier food advertisements targeting youth were shown in children’s non-peak time and morning during news and entertainment programmes. Product uniqueness, humour, and fun were the most frequent primary persuasive techniques. Most advertisements showed a high use of brand logos, product images, and premium offers. In conclusion, Portuguese children and adolescents are potentially exposed to large numbers of unhealthy food advertisements on television, despite marketing regulation and restriction policies.

## 1. Introduction

In recent decades, childhood obesity has increased dramatically, posing a threat to children’s and adolescents’ health [1]. Children who are overweight and obese are more likely to live with obesity in adulthood and to develop chronic non-communicable diseases such as diabetes, cardiovascular disease, high blood pressure, and respiratory problems, and they are additionally associated with social exclusion problems, anxiety, and depression [2]. Globally, in 2016, 40 million children under the age of 5 years and over 330 million children and adolescents between 5 and 19 years were overweight or obese [3]. In Portugal, according to the Childhood Obesity Surveillance Initiative (COSI) 2019, around 29.7% of children aged between 6 and 8 present as overweight, and 11.9% present as obese [4]. 

Food marketing is a key characteristic of the food environment—heavy marketing of energy-dense foods and fast-food outlets was recently pointed out by the World Health Organization (WHO) and the Food and Agriculture Organization of the United Nations (FAO) as a “probable” cause for the increasing prevalence of childhood obesity [5]. 

**Figure 1:**
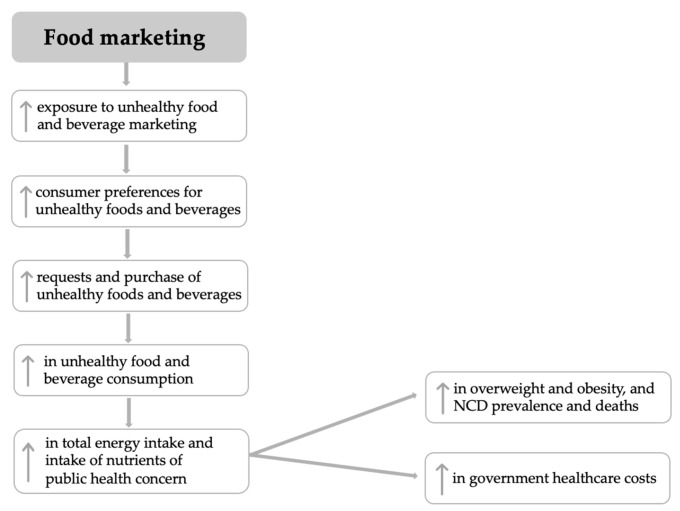
Impact of food marketing to children; Adapted from UNICEF, 2021 [11]. Arrows pointing up (↑) represent an increase.

The impact of unhealthy food marketing on children’s food-related preferences, attitudes, and behaviours has been widely studied and demonstrated through several research reviews [6,7,8,9,10]. The pathway by which food marketing impacts children’s health is described in Figure 1, adapted by UNICEF’s policy brief on the marketing of unhealthy foods and non-alcoholic beverages to children [11]. Children and adolescents are constantly exposed to large volumes of unhealthy food marketing through a variety of channels [7]. Evidence shows that there are four times more advertisements on television for foods/beverages that should not be permitted than for permitted foods/beverages [12]. Most advertised products are classified as unhealthy—sugary breakfast cereals, savoury snacks, cakes and sweets, soft drinks, and fast-food (commonly referred to as the “Big Five”)—whereas vegetables and fruits, on the other hand, are significantly underrepresented [6]. Research recognizes that exposure to unhealthy food and beverage advertising has a negative impact on children’s preferences for energy-dense foods [13], influencing their request, purchase, and consumption patterns for unhealthy food products high in fats, sugars, and salt [6,8,14]. Children are easy targets for marketing strategies and are the main influencers of family food purchases (particularly towards advertised products) [13,15]. Consistent evidence shows that exposure to food marketing increased children’s total energy intake, particularly due to a higher consumption of energy-dense and low-in-nutrient foods [7,8,10,13]. For example, a recent systematic review found that exposure to 4.4 min of television food advertising increased children’s food consumption by 60.0 kcal, on average [16]. Moreover, results from the same study show that children who are overweight or obese consumed 57% more calories (45.6 kcal) than children with healthy weight, suggesting that children who are overweight are more vulnerable to the effects of unhealthy food marketing. Finally, exposure to unhealthy food marketing contributes to unhealthy diets and poor health in children, leading to weight gain over time and increased risk of being overweight and obese [6,8,14]. 

A key recommendation to protect children from the marketing of food and drinks high in fat, salt, or sugar (HFSS) is the implementation of mandatory policies to restrict food marketing to which children are exposed [17]. In addition to restricting children’s exposure to unhealthy food marketing, WHO recommends that “all policy frameworks should include a monitoring system to ensure compliance with the objectives set out in the national policy” [18].

In Portugal, since April 2019, Law no. 30/2019 has been implemented, which applies restrictions to HFSS food advertising aimed at children under 16 years of age [19]. The law covers advertisements broadcasted in the 30 min before and after programmes targeted at children or for which a minimum of 25% of the audience includes children under 16 years old—it includes television programs and services, on-demand audio-visual communication services, and radio, including advertising in the respective breaks of these programs, as well as digital marketing restrictions. It is incumbent upon the Portuguese Directorate General of the Consumer to ensure the monitoring of compliance with this law and breaches are punished with fines of €1750 to €3750 if committed by a natural person (that is, an individual human being), or 3500€ to 45,000€ if committed by a legal person (for example, agencies, corporations, associations, or committees). Accordingly, the Directorate-General for Health identified, in accordance with the WHO recommendations for the definition of the nutritional profile of these foods [20], the values that should be considered in the identification of HFSS foods, published through Dispatch no. 7450-A/2019 [21]. The implementation of legislation in this area involves a prior definition of a monitoring system. Thereupon, since 2016, the WHO has proposed a protocol to provide the basis for monitoring and quantifying the extent and nature of children’s exposure to the marketing of HFSS foods, via television (TV) and the internet [22].

According to the National Food, Nutrition and Physical Activity Survey (IAN-AF) in 2015–2016, 37% of Portuguese children and adolescents between 3 and 14 years spend more than 2 h per day on weekdays watching television, and this prevalence increases to 71% on weekends [23]. Hence, although the constant growth of digital marketing is a big concern and young people are ever more engaged in the internet and social media platforms [24], traditional media, such as TV, should still be considered a major source of food marketing exposure to children. 

It is, therefore, essential to continuously monitor compliance with Law no. 30/2019 on food marketing restrictions in Portugal, through the analysis of food advertising aimed at children. 

## 2. Materials and Methods

### 2.1. Aim

The main aim of this study was to assess the potential exposure and power of food advertisements aimed at children, broadcasted on Portuguese TV channels. Exposure is defined as “the reach and frequency of the marketing message”, whereas power is “the creative content, design, and execution of the marketing message” [17].

### 2.2. Television Sampling

The present study was conducted according to the standardized methodology (sampling, recording, and coding of the advertisements) of the WHO protocol “Monitoring food and beverage marketing to children via television and the Internet” [22]. This observational study followed an adapted version of this protocol, adjusted to the Portuguese legislation [19,21].

Television transmission was recorded from four generalist and free-access Portuguese channels (RTP1, RTP2, SIC, and TVI) between 6 am and 10 pm hours on two weekdays (Tuesday and Friday) and two weekend days (Saturday and Sunday) in November 2021, avoiding national holidays, major sporting competitions, special events, or other festive seasons. Children’s channels were excluded from the analysis since previous studies have shown that there are no advertisements on food products in these channels [25]. After the video acquisition, the material was stored and scanned for advertisements. 

### 2.3. Commercial Coding

Recorded television data were analysed according to the WHO proposed tool and coding templates [22], adapted to the Portuguese legislation. Opening and closing credits, closed captioned acknowledgments, brief sponsorship announcements, and content promotions that appear later in the same programme were not considered advertisements. 

Details of the TV advertisements were grouped into 2 categories: the exposure variables (quantity, frequency, and reach of marketing communications for unhealthy foods to children) and the power variables (prevalence of specific marketing techniques used). Potential exposure was analysed for all advertisements (including food and non-food), whereas power variables were only accessed for food advertisements.

### 2.4. Exposure Variables 

All advertisements broadcasted by the selected TV channels were coded. Coding categories included the country, channel, date, day of the week, programme name, programme category (e.g., comedy, drama, movie; etc.), programme start time, time slot of the advertisement, whether the programme occurs during peak or non-peak children’s viewing time, whether the programme occurs between or within the programme (i.e., during the programmes breaks), and advertised product type (e.g., food and beverages, clothes/shoes, education, entertainment; etc.). Five additional categories of advertised product type were added to adapt to country-specific trends in advertising, namely: supermarkets and food delivery; lottery; real estate agencies; mobility/medical equipment, and gadgets (including smartphones, watches…). 

Peak children’s viewing times are defined as the period of time in which the number of children watching television is greater than 25% of the maximum child audience that is likely to be watching [22]. Due to the lack of this data, the time slot between 5:00 p.m. and 8:00 p.m. was considered as peak viewing time for children, according to previous studies [26,27]; other viewing times were designated as “non-peak”. 

For food and beverage advertisements additional coding variables were accessed, namely the food-product brand name, detailed description of the food product, nutrient profile food-category code (e.g., chocolate, beverages, cheese, etc.), nutritional information (including total fat, saturated fat, total sugars, added sugars, non-sugar sweeteners, salt, energy, and fibre) and whether the marketing is permitted according to the Portuguese Nutrient Profile Model (NPM) outlined by the Portuguese Directorate-General of Health [21].

### 2.5. Power Variables

The power of persuasive techniques was only assessed for food and beverage advertisements and was divided into two categories: (i) primary persuasive techniques and (ii) secondary persuasive techniques.

Primary persuasive techniques represented the main visual and auditory appeal that advertisements displayed to persuade the consumer (e.g., quantity, convenience, taste, health/nutrition, energy, price, etc.). As for secondary persuasive techniques, these were represented by additional techniques used in the advertisement to increase engagement with the advertised products. Five sub-categories for secondary persuasive techniques were evaluated:

1. Increased advertisement engagement: dynamic audio-visual components;
2. Increased brand engagement and brand synergy: including brand equity characters, licensed characters, cartoon characters, and celebrity endorsers;
3. Increased awareness of the brand and website: including the website address provided and links to social media platforms;
4. Consumption norms, brand, and product imagery: including brand logos, images of the product or packaging, child or child-like characters, and premium offers;
5. Health-related imagery or messaging: including health claims (e.g., low fat/fat free, sugar free, organic, etc.), physical activity depicted, and disclaimers (e.g., part of a balanced/healthy diet, enjoy in moderation, etc.).

The targeting content of the advertisement was also evaluated—children, children and teenagers, teenagers, teenagers and adults, adults, the elderly, and the whole family. The following criteria were considered in the identification of food advertisements aimed at children and teenagers: the presence of cartoons, animations, or children, and informal language aimed at a young public.

Also, additional variables in accordance with Portuguese legislation [19] were assessed:

- if the advertising creates a sense of urgency or need to consume the product;
- if the advertising conveys the idea of facilitating its acquisition, minimizing its costs;
- if the advertising conveys the idea of benefit in its exclusive or exaggerated consumption, compromising the appreciation of a varied and balanced diet and healthy lifestyle;
- if the advertising associates the consumption of the product with the acquisition of status, social success, special skills, popularity, success, or intelligence;
- if the advertising communicates characteristics of high energy content, salt, sugar, and saturated and trans fatty acids in food and beverages as health benefits, omitting the harmful effects of these high levels.

### 2.6. Food and Beverage Products Coding

When more than one food product appeared in the advertisement, only the most dominant product (appearing for most of the commercial’s time) was considered. When more than one product received equal attention, all the products were considered—in such cases, if one of the products was not in accordance with the Portuguese NPM, the advertisements’ food content was then considered as not permitted. 

To obtain the nutritional information (including total fat, saturated fat, total sugars, added sugars, non-sugar sweeteners, salt, energy, and fibre) required to correctly classify advertised products, company websites (Continente^®^ Sonae, Porto, Portugal; Auchan^®^ Retail Portugal, Lisbon, Portugal and ElCorteInglês^®^ S.A., Lisbon, Portugal) were accessed and the ingredients labels on product packaging were checked on December 2021.

### 2.7. Statistical Analyses

Statistical analyses were performed using the IBM^®^SPSS^®^ Statistics software (IBM Corp. Released 2020. IBM SPSS Statistics for Windows, Version 27.0. Armonk, NY, USA: IBM Corp.) after coding the data in the WHO television coding templates, and the significance level was fixed at 0.05. Descriptive analyses were presented as means (±SD) for continuous variables and as frequency distributions (%) for categorical variables. Proportions were compared using the Chi-square test to assess the differences between all food and beverage advertisements and food and beverage advertisements targeted at youth, according to different variables (e.g., if advertisements occurred during children’s peak viewing time).

## 3. Results

### 3.1. Overall Marketing Extent

During the recording days on the four generalist and free-access Portuguese channels, we obtained 256 h of TV broadcasting, with a total of 5272 advertisements transcribed. Overall, the top five advertised products on Portuguese television were retailing and mail order, including catalogues (*n* = 646, 12.2%), supermarkets and food delivery (*n* = 633, 12%), food and beverages (*n* = 590, 11.2%), channel promotions (*n* = 552, 10.5%), and pharmaceuticals (*n* = 437, 8.3%). 

As for food and beverage advertisements, the proportions of advertisements ranged across TV channels from 4.6% (*n* = 27) on RTP1 to 40.3% (*n* = 238) on SIC and 55.1% (*n* = 325) on TVI. There was no advertising for food and beverages on RTP2.

### 3.2. Food and Beverage Marketing Extent of Exposure 

Across all TV channels, most food and beverage advertisements targeted all ages/family (44.6%), followed by adults (25.9%). Children and teenagers were targeted by a total of 131 food and beverage advertisements (22.2%). 

**Figure 2:**
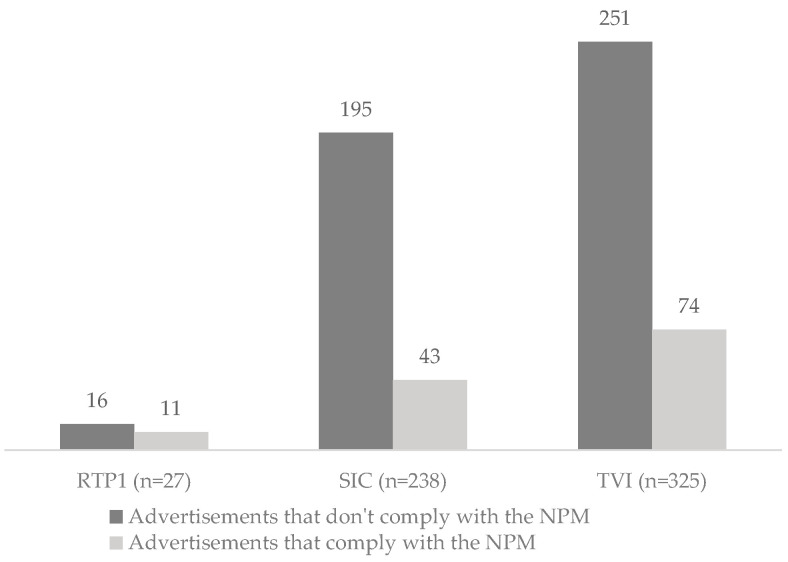
Number of food and beverage TV advertisements according to compliance with the Portuguese NPM (*p* = 0.020).

Among all food and beverage advertisements, 78.3% did not meet the nutritional profile outlined by the Portuguese Directorate-General of Health [21]. The prevalence of food and beverage advertisements according to the compliance with the nutritional profile outlined by the Portuguese Directorate-General of Health is described by TV channel in Figure 2.

The prevalence of food and beverage advertisements according to food category is described in Table 1. Ready-made convenience foods (mainly pizzas and hamburgers from fast-food companies) were the most advertised category (25.9%), followed by chocolate and bakery products (23.6%), others—including coffee and teas—(9.5%) and soft drinks (9.3%). No advertisements on fruit, vegetables, or legumes were registered and only a small percentage of meat/fish/eggs advertisements was found. Regarding the food and beverage advertisements targeting children and teenagers, chocolate and bakery products were the most advertised food category (42%), followed by soft drinks (26.7%) and yoghurt and fermented milk (16%)—none of the food and beverage advertisements met the nutritional profile outlined by the Portuguese Directorate-General of Health [21].

Characteristics of food and beverage advertisements according to different factors are described in Table 2. When comparing all food advertisements and those targeting children and teenagers, according to different factors, the differences were statistically significant for programme category (*p* < 0.001), peak/non-peak time (*p* = 0.023), and time slot (*p* = 0.002). As for the day of the week and if the advertisement occurred within or between programme, no differences were found (*p* = 0.072 and *p* = 0.602, respectively).

Most of the food and beverage advertisements, as well as those targeting children and teenagers, occurred during news/commentary programmes (42.4% and 42.0%, respectively) and entertainment/variety shows (34.6% and 35.9%, respectively)—only about 7% of the advertisements targeting youth occurred during children’s shows. Just over half of all food and beverage advertisements (52.7%) occurred during the weekdays and about a third of all advertisements (*n* = 173, 29.4%) occurred during children’s peak viewing time. As for the food and beverage advertisements directed at youth, the majority (54.2%) occurred during the weekend days and only 21.4% on children’s peak viewing time. Most of the advertisements occurred within the programmes. Most of the food and beverage advertisements occurred during the afternoon period (*n* = 202, 34.2%), and most of the advertisements targeting children and teenagers occurred during the morning period (*n* = 56, 42.7%). 

**Figure 3:**
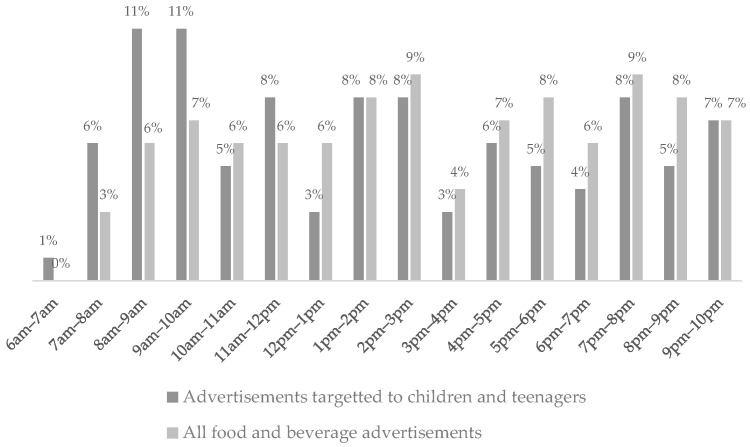
Time slot percentage of food and beverage TV advertisements (*p* = 0.018).

Regarding advertisement time slots, most of the advertisements occurred between 2 pm–3 pm (9%) and 7 pm–8 pm (9%), and most of the advertisements targeting children and teenagers occurred between 8 am–9 am (11%) and 9 am–10 am (11%) (Figure 3). 

### 3.3. Marketing Persuasive Techniques

**Figure 4:**
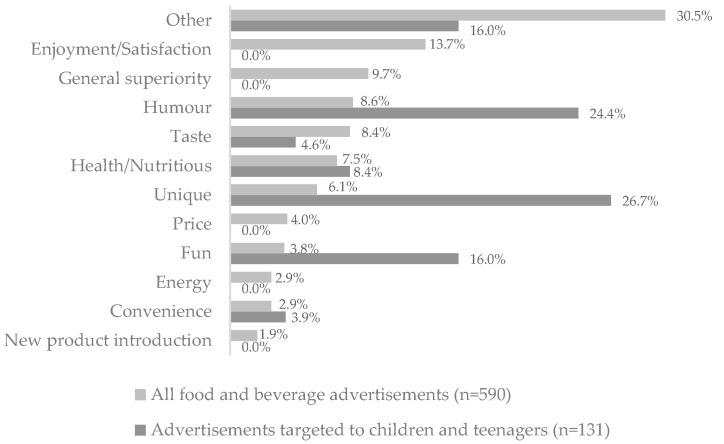
Percentage of primary persuasive techniques used in food and beverage TV advertisements (*p* < 0.001).

Among primary persuasive techniques, the “other” category was the most frequently used in all food and beverage advertisements (*n* = 180, 30.5%). This category stood out when the advertisements focused on sustainability matters and environmental protection, vegetable protein choices, and fair-trade labels. “enjoyment/satisfaction” (*n* = 81, 13.7%) and “general superiority” (*n* = 57, 9.7%) were the second and third most used primary persuasive techniques. Considering food and beverage advertisements targeted to a young public, “unique” was the most used primary persuasive technique (*n* = 35, 26.7%), followed by “humour” (*n* = 32, 24.4%) and “fun” (*n* = 21, 16.0%) and “other” (*n* = 21, 16.0%) (Figure 4).

Regarding secondary persuasive techniques, “image of the product/packaging” was the most often used marketing technique (*n* = 586, 99.3%), and “brand logo” was the second most used technique in all food and beverage advertisements, being displayed on screen in 89.2% (*n* = 526) of the advertisements. Dynamic audio-visual components and brand equity characters were displayed on screen in 34.9% (*n* = 206) and 9% (*n* = 53) of all food and beverage advertisements, respectively. As for licensed and cartoon characters, these were absent in the totality of the advertisements. Regarding celebrity endorsers, only 15 advertisements had the presence of an entertainment celebrity (2.5%) and 9 advertisements of a sports celebrity (1.5%). Only 5% of the advertisements presented health claims, such as “sugar free”, “no added sugars/less sugar” and “low calorie/light” and only 9 advertisements (1.5%) used the “part of a balanced/complete/nutritious breakfast or meal” disclaimer (Table 3).

Regarding the advertisements targeting children and teenagers, the “image of the product/packaging” was displayed in all advertisements and the “brand logo” in 96.9% (*n* = 127) of the advertisements, being the two most used marketing techniques. The website address was present in 13.0% (*n* = 17) of the advertisements and no links to social media platforms were displayed. Dynamic audio-visual components and brand equity characters were displayed on screen in 42.7% (*n* = 56) and 25.2% (*n* = 29) of food and beverage advertisements targeting youth, respectively. No health claims or disclaimers were presented in any of the food and beverage advertisements targeting youth, neither was physical activity depicted. Also, licensed characters, cartoon characters, and celebrity endorsers were absent (Table 3).

Marketing techniques were more often used in advertisements aimed at children and teenagers, and differences were statistically significant for dynamic audio-visual components (*p* = 0.033), brand equity characters (*p* < 0.001), brand logos (*p* = 0.001), and child characters (*p* < 0.001).

### 3.4. Additional Variables in Accordance with Portuguese Legislation

Regarding the additional variables in accordance with the Portuguese legislation, 9.5% (*n* = 56) of all beverage and food product advertisements created a sense of urgency or need to consume the product, and about 6% of the advertisements associated the consumption of the product with the acquisition of status, social success, special skills, popularity, success, or intelligence. About 10% of the advertisements conveyed the idea of facilitating its acquisition, and minimizing its costs, mostly when it refers to the possibility of home delivery (Uber eats^®^ and Glovo^®^), free delivery, and discounts (Table 4). Regarding the advertisements targeting children and teenagers, 26.7% (*n* = 35) associated the consumption of the product with the acquisition of status, social success, special skills, popularity, success, or intelligence, and only 7.6% of the advertisements created a sense of urgency or need to consume the product (Table 4).

## 4. Discussion

Present findings indicate that Portuguese children might be largely exposed to food and beverage advertisements on television, which are mainly classified as unhealthy. In our sample, consistent with past research [26,28,29,30,31,32,33], food and beverage were among the top 3 advertised products, accounting for 11.2% of total advertisements. In general, the percentage of food advertisements found in this study was lower than results from past studies, ranging from 12.8% in the United Kingdom [28] to 25% in both Australia and China [26,31] and similar to the results obtained from a study conducted in Portugal in 2020 (10.4%) [25]. 

The most frequently advertised products, overall, were ready-made and convenience foods, chocolate and bakery products, others, and soft drinks. Yet, when analysing the most frequently advertised products to children and teenagers, chocolate and bakery products comprised the most advertised food category, followed by soft drinks, yoghurt, and fermented milk. A possible explanation for this difference is that an older audience (adults and families) is more likely to buy these ready-made and convenience foods, whereas a younger public more often chooses food like chocolates and soft drinks, which are more easily accessible. In Russia, Kontsevaya et al. [32] found beverages, chocolate and confectionery, and ready-made and convenience foods to be among the most advertised products overall. Also, in the study conducted by Kelly and colleagues [29], fast-food restaurant meals and chocolate and confectionery were the two most advertised products across the 11 countries considered. Our study also found that there were no food advertisements that promoted fruits or vegetables, similar to a previous study in China [26]. 

Of the total advertisements analysed, about one-fifth were considered to be aimed at children and teens, a higher prevalence than the one found in the study conducted in Portugal in the previous year (18.6%) [25]. However, 44.6% of the food and beverage advertisements were considered to be aimed at families, which means that these advertisements could also be directed at children and adolescents and therefore also aim to influence their dietary habits. Our research also provides some insight into the fragilities of the Portuguese Law no. 30/2019 on food marketing restrictions that are worth further discussion. According to the WHO framework for implementing the set of recommendations on the marketing of foods and non-alcoholic beverages to children, defining child-directed advertising as TV advertising during programmes with an audience of (in the case of Portugal) at least 25% children under the age of 16 does not cover programmes for both children and adults or other programmes popular among children but not made especially for them, such as sporting events or family shows [17]. Future restrictions should encompass these ambiguity issues regarding the concept of “marketing to children” and cover mixed audiences’ programmes. 

Despite Portuguese marketing regulations, none of the food advertisements aimed at children and adolescents met the NPM defined by the Directorate-General of Health [19,21]. Consistent with these results, Kontsevaya and colleagues [32] and Cossenza-Quintana and colleagues [34] recently showed that 64.2% and 85% of food advertisements should not be permitted to be advertised to children according to the WHO NPM, respectively. 

Non-peak children’s viewing time and the morning and afternoon time slots (rather than meal time—lunch and dinner) were the periods during which food advertisements for children appeared most frequently. Contrary to our results, previous research in the United Kingdom and Australia showed a significantly higher percentage of food advertisements during children’s peak viewing times, compared to non-peak times [28,31]. Also, more food advertisements targeting youth were shown during weekend days, compared to weekdays. According to the IAN-AF results [22], television use among children and adolescents was higher during the weekend days. Nonetheless, considering that there were no significant differences in the amount of non-permitted food advertisements transmitted throughout the day, policies and regulations should be focused on the restriction of food advertisements regardless of peak hours.

In this paper, we also aimed to analyse the marketing strategies associated with the advertised products. Because all the food advertisements aimed at children were for foods that did not meet the NPM, we were unable to make a comparative analysis regarding marketing persuasive techniques of unhealthy vs. healthy food products. Nevertheless, in the current study, product uniqueness, humour, and fun were the most frequently used primary persuasive techniques in advertisements considered to be aimed at children. In 2014, Jenkin et al. [35] performed a systematic review and found that the main persuasive techniques used by food advertisements targeted to a young public (among 38 studies) were premium offers, promotional characters, nutrition or/and health-related claims, taste, and fun. In fact, regarding secondary persuasive techniques, in our study, premium offers were displayed on screen in 27.5% of the advertisements targeting children, and brand equity characters were present in 25.2% of the advertisements. Similarly, Kelly et al. found that, across 11 countries considered in their study, 12% of food advertisements contained premiums and 23% contained promotional characters [29]. The use of these techniques varied between countries but was used more often in advertisements for noncore food products. Moreover, evidence confirms that persuasive marketing techniques, such as premium offers and promotional characters influence children’s preferences and purchasing requests [13]. In addition to these marketing techniques, our results showed, overall, high use of dynamic audio-visual components, brand logos, and the image of the product. These marketing strategies influence the “power” of marketing communications, i.e., the creative content, design, and execution of the marketing message [17].

Evidence shows that exposure to food advertising increases dietary intake in all children, but the increase is higher in overweight and obese children [13]. Additionally, children’s food preferences and purchase requests are massively influenced by TV food advertising [36], particularly their preference for energy-dense foods during or shortly after exposure to advertisements [10]. Thus, restricting unhealthy food marketing may be an effective strategy to reduce childhood obesity rates. 

One of the main strengths of this study was the application of the WHO protocol and templates, which highly impacts the methodological quality of the study and allows comparability with other countries. The large sample size of the advertisements (*n* = 5272) and recorded hours (*n* = 256), as well as the wide set of variables assessed, are additional strengths. In addition, for advertisements with multiple products, all products were coded, and nutrition information was collected, excluding the possibility of potentially ignoring the presence of additional unhealthy products. 

In terms of policy recommendations, our paper highlights that policies should be as comprehensive and broad as possible [17]. Food marketing restrictions should be time-based, rather than depending on the type of program or children’s audience. Furthermore, according to a recent study conducted in Chile, policies should “include broadly defined content-, placement-, and time-based restrictions” in order to effectively protect children from unhealthy food marketing [37]. 

Our study is not without limitations: firstly, our concept of peak viewing time might not be accurate enough since it is based on previous studies and not on the yearly television audience data for this age group, as we did not have access to that data. Moreover, we did not measure actual children’s exposure to food advertising, hence the fact that this study does not necessarily allow us to assess whether or not Portuguese Law no. 30 is being violated. Other monitoring approaches would be necessary to conduct that research, such as survey data on audience size and characteristics, surveys of children’s use of TV and other forms of media, and also surveys to assess exposure to marketing [17]. Furthermore, the four selected channels were only a proportion of what children are potentially exposed to, as children could watch other TV channels. Second, we did not register the advertisements’ duration, which would be an important aspect as some authors remark that it can impact children’s food choices. Third, we did not measure product placement in children’s movies, which is an important aspect of food marketing on television and has been shown to have a significant effect on children’s snack and drink consumption [38]. Also, as programme sponsorship is not included, our findings are likely to underestimate the full extent of food promotion on TV. Another important limitation of our study is the researcher’s subjectivity in the evaluation of some of the variables under study. More extensive research is needed to assess the impact of these food and beverage advertisements on children’s health outcomes, specifically childhood obesity. To overcome the above-mentioned limitations, future studies in Portugal should replicate these methodologies in larger-scale analyses, including more marketing techniques (such as product placement and programme sponsorship) and more television channels, but also explore other channels of marketing to children, such as internet, outdoor, and digital marketing.

This study is part of the food marketing monitoring work that is being developed with the support of WHO Europe for the member states of the WHO European Region and within the scope of the Best ReMaP Joint Action. In the future, it will be possible to frame them in a more comprehensive context, together with the results of studies carried out for other European countries. 

## 5. Conclusions

Our findings suggest that, despite marketing regulation and restriction policies currently in place in Portugal, there is still a high percentage of food advertisements with potentially child-directed content on television, and children and adolescents are likely exposed to large numbers of unhealthy food advertisements on television. 

Continuous monitoring is essential to ensure compliance with the policies in place as well as to support the development or update of these policies. Also, more research is needed in this area to evaluate policy impact, particularly on children’s exposure to marketing and on the effect of marketing on children’s attitudes, beliefs, and preferences and on their actual food consumption, dietary patterns, and health. 

These findings may be of interest to researchers, public health professionals, and policymakers, as they provide important insights for future research regarding the development, update, and better monitoring of policies to protect children from unhealthy food marketing. Furthermore, our study offers important information for advertisers, as it highlights the importance of designing responsible advertising strategies that promote a healthy lifestyle. And finally, it highlights parents and caregivers’ crucial role in shaping children’s dietary behaviours.

## Figures and Tables

**Table 1:** Percentage of food and beverage TV advertisements, by food category.

	All Food and Beverage Advertisements	Advertisements Targeted to Children and Teenagers
Food Category		
Chocolate, bakery products, energy bars, sweet spreads, and toppings	139 (23.6%)	55 (42.0%)
Ready-made and convenience foods and composite dishes	153 (25.9%)	4 (3.1%)
Bread, bread products, and crisp breads	1 (0.2%)	---
Meat, fish, and eggs	11 (1.9%)	---
Processed meat, poultry, fish, and similar	1 (0.2%)	---
Cakes and other pastries	21 (3.6%)	5 (3.8%)
Other	56 (9.5%)	---
Snacks	1 (0.2%)	---
Milk	29 (4.9%)	---
Soft drinks	55 (9.3%)	35 (26.7%)
Ice-cream	16 (2.7%)	---
Breakfast cereal	20 (3.4%)	11 (8.4%)
Yoghurt, fermented milk, and similar	48 (8.1%)	21 (16.0%)
Cheese and analogues	39 (6.6%)	---
Total	590	131

**Table 2:** Percentage of food and beverage TV advertisements, according to different factors.

	All Food and Beverage Advertisements	Advertisements Targeted to Children and Teenagers	*p*-Value
Advertisement permitted according to the Portuguese NPM			-
Yes	128 (21.7%)	---	
No	462 (78.3%)	131 (100%)	
Programme category			<0.001
News/Commentary	250 (42.4%)	55 (42.0%)	
Entertainment/Variety show	204 (34.6%)	47 (35.9%)	
Talk show	78 (13.2%)	12 (9.2%)	
Children’s show	12 (2.0%)	9 (6.9%)	
Soap opera	30 (5.1%)	4 (3.1%)	
Drama	6 (1.0%)	1 (0.8%)	
Other	10 (1.7%)	3 (2.1%)	
Children’s peak viewing time			0.023
Peak viewing time (5 pm–8 pm)	173 (29.3%)	28 (21.4%)	
Non-peak	417 (70.7%)	103 (78.6%)	
Timeslot			0.002
Morning	168 (18.5%)	56 (42.7%)	
Lunch time (12 pm–2 pm)	82 (13.9%)	15 (11.5%)	
Afternoon	202 (34.2%)	34 (26.0%)	
Dinner time (7 pm–9 pm)	96 (16.3%)	17 (13.0%)	
Night	42 (7.1%)	9 (6.9%)	
Day of the week			0.072
Weekday	311 (52.7%)	60 (45.8%)	
Weekend day	279 (47.3%)	71 (54.2%)	
If the advertisement occurred within or between the programmes			0.602
Within	394 (66.8%)	85 (64.9%)	
Between	196 (33.2%)	46 (35.1%)	

*p*-value was calculated using the Chi-square test.

**Table 3:** Secondary persuasive techniques used in food and beverage TV advertisements.

	All Food and Beverage Advertisements	Advertisements Targeted to Children and Teenagers	
Secondary Persuasive Techniques			*p*-Value
Dynamic audio-visual components			0.033
Absent	384 (65.1%)	75 (57.3%)	
Displayed on screen	206 (34.9%)	56 (42.7%)	
Brand equity characters			<0.001
Absent	537 (91%)	98 (74.8%)	
Displayed on screen	53 (9.0%)	29 (25.2%)	
Licensed characters			
Absent	590 (100%)	131 (100%)	---
Cartoon characters			---
Absent	590 (100%)	131 (100%)	
Celebrity endorsers			-
Absent	566 (95.9%)	131 (100%)	
Entertainment celebrity	15 (2.5%)	---	
Sports celebrity	9 (1.5%)	---	
Website address			0.349
Absent	498 (84.4%)	114 (87.0%)	
Displayed on screen	92 (15.6%)	17 (13.0%)	
Links to social media platforms			-
Absent	574 (97.3%)	131 (100%)	
Present	16 (2.7%)	---	
Brand logos			0.001
Absent	64 (10.8%)	4 (3.1%)	
Displayed on screen	526 (89.2%)	127 (96.9%)	
Image of product/packaging			-
Absent	4 (0.7%)	---	
Displayed on screen	586 (99.3%)	131 (100%)	
Child character			<0.001
Absent	536 (90.8%)	94 (71.8%)	
Present	54 (9.2%)	37 (28.2%)	
Premium offers			<0.001
Absent	519 (88.0%)	95 (72.5%)	
Present	71 (12.0%)	36 (27.5%)	
Health claims			-
None	561 (95.1%)	131 (100%)	
Other	8 (1.4%)	---	
Sugar free	7 (1.2%)	---	
No added sugars/less sugar	10 (1.7%)	---	
Low calorie/light	3 (0.5%)	---	
Whole grain/Whole wheat	1 (0.2%)	---	
Physical activity			-
None depicted	573 (97.1%)	131 (100%)	
Depicted	17 (2.9%)	---	
Disclaimers	581 (98.3%)	131 (100%)	-
None			
Part of a balanced/complete/	9 (1.5%)	---	
nutritious breakfast or meal			

*p*-value was calculated using the Chi-square test.

**Table 4:** Percentage of food and beverage TV advertisements, according to variables foreseen in the Portuguese Legislation.

	All Food and Beverage Advertisements	Advertisements Targeted to Children and Teenagers	*p*-Value
Urgency in consumption			0.411
Yes	56 (9.5%)	10 (7.6%)	
No	534 (90.5%)	121 (92.4%)	
Facilitation			-
Yes	56 (9.5%)	---	
No	534 (90.5%)	131 (100%)	
Status			<0.001
Yes	36 (6.1%)	35 (26.7%)	
No	554 (93.9%)	96 (73.3%)	

*p*-value was calculated using the Chi-square test.

## Data Availability

The datasets generated and/or analysed during the current study are not publicly available but are available under reasonable request to the corresponding author.
